# Balloon dilatation is superior to CO_2_ laser excision in the treatment of subglottic stenosis

**DOI:** 10.1007/s00405-023-07926-w

**Published:** 2023-03-24

**Authors:** Eleftherios Ntouniadakis, Josefin Sundh, Anders Magnuson, Mathias von Beckerath

**Affiliations:** 1 Department of Ear Nose and Throat, Faculty of Medicine and Health, Örebro University, 70182 Örebro, Sweden; 2 Department of Respiratory Medicine, Faculty of Medicine and Health, Örebro University, 70182 Örebro, Sweden; 3 Clinical Epidemiology and Biostatistics, School of Medical Sciences, Faculty of Medicine and Health, Örebro University, 70182 Örebro, Sweden; 4 Department of Clinical Sciences, Intervention and Technology, Karolinska Institute, Karolinska University Hospital, Stockholm, Sweden; 5 Ear Nose and Throat Department, Örebro University Hospital, Södra Grev Rosengatan, 701 85 Örebro, Sweden

**Keywords:** Subglottic stenosis, Balloon dilatation, CO_2_ laser, Endoscopic treatment

## Abstract

**Introduction:**

Endoscopic treatment of subglottic stenosis (SGS) is regarded as a safe procedure with rare complications and less morbidity than open surgery yet related with a high risk of recurrence. The abundance of techniques and adjuvant therapies complicates a comparison of the different surgical approaches. The primary aim of this study was to investigate disease recurrence after CO_2_ laser excisions and balloon dilatation in patients with SGS and to identify potential confounding factors.

**Materials and methods:**

In a tertiary referral center, two cohorts of previously undiagnosed patients treated for SGS were retrospectively reviewed and followed for 3 years. The CO_2_ laser cohort (CLC) was recruited between 2006 and 2011, and the balloon dilatation cohort (BDC) between 2014 and 2019. Kaplan‒Meier and multivariable Cox regression analyzed time to repeated surgery and estimated hazard ratios (HRs) for different variables.

**Results:**

Nineteen patients were included in the CLC, and 31 in the BDC. The 1-year cumulative recurrence risk was 63.2% for the CLC compared with 12.9% for the BDC (HR 33.0, 95% CI 6.57–166, *p* < 0.001), and the 3-year recurrence risk was 73.7% for the CLC compared with 51.6% for the BDC (HR 8.02, 95% CI 2.39–26.9, *p* < 0.001). Recurrence was independently associated with overweight (HR 3.45, 95% CI 1.16–10.19, *p* = 0.025), obesity (HR 7.11, 95% CI 2.19–23.04, *p* = 0.001), and younger age at diagnosis (HR 8.18, 95% CI 1.43–46.82, *p* = 0.018).

**Conclusion:**

CO_2_ laser treatment is associated with an elevated risk for recurrence of SGS compared with balloon dilatation. Other risk factors include overweight, obesity, and a younger age at diagnosis.

## Introduction

Subglottic stenosis (SGS) is a rare condition of mucosal scarring, compromising the extrathoracic part of the tracheal airway below the vocal folds. An inflammatory response leading to fibrosis can be triggered by prolonged intubation or tracheostomy, gastroesophageal reflux disease (GERD), or autoimmune conditions, such as vasculitis, sarcoidosis, and relapsing polychondritis [1]. The idiopathic type of SGS is considered to be very rare with an incidence of up to 1:200,000, affecting otherwise healthy perimenopausal females of Caucasian origin [1, 2]. Since SGS presents with common and relatively unspecific symptoms, such as exertional dyspnea, wheezing, chronic cough, or dysphonia, it is frequently misinterpreted as a difficult-to-treat lower airway obstruction resulting in a diagnostic delay of up to 2 years; thus, occasionally manifesting with stridor at rest [3].

Given that recurrence of SGS is regarded as the natural course of the condition, the main treatment goal is to restore durable airway patency without the need for tracheostomy. Open surgical procedures are considered to have the lowest incidence of recurrence; thus, a chance for a permanent treatment. However, these procedures are quite demanding with respect to institutional resources and are associated with increased perioperative and postoperative morbidity in terms of voice and swallowing deterioration [4–6]. Endoscopic techniques are low-risk, voice-sparing procedures that are safe to perform in an outpatient surgery setting; thus, have high patient acceptance [7–9]. However, they are considered to have a significantly higher recurrence rate than open surgery, reported to be approximately 30% within 1 year postoperatively, 50% within 2 years, and 80% within 3 years [10, 11]. Resection of quadrants of the fibrotic tissue with carbon dioxide (CO_2_) laser and balloon dilatation alone or following cold knife incisions in the stenotic part of the airway have frequently been used, among others, as the endoscopic treatment of SGS [12]. The rarity of SGS combined with the different types and concepts of endoscopic procedures, the divergence of volumes and resources in different institutions, and other unmeasured confounding factors leading to a selection bias make the comparison of these two techniques complicated [11, 13].

The aim of this study was to describe the disease characteristics of the patient cohort treated for SGS in our institution, a tertiary referral center in Sweden, to retrospectively assess whether balloon dilatation is a superior treatment compared to CO_2_ laser excision of the scar tissue, and to identify potential confounding factors in terms of time to disease recurrence.

## Materials and methods

### Study subjects

Previously undiagnosed adult patients treated primarily for isolated SGS at the Örebro University Hospital, a tertiary academic referral center in Sweden, between 1 January 2006 and 31 December 2019 were identified based on a retrospective chart review of relevant ICD-10 codes, in particular J38.6, J95.5, and J95.8. Patients with SGS caused by malignant tumors, external compression of the airway, or a damaged laryngotracheal cartilaginous framework, and those previously treated for stenosis in the laryngotracheal part of the airway, or with multilevel and distal tracheal strictures, were excluded from the study.

### Surgical techniques

From the early 1990s until 2011, patients with SGS had traditionally been treated with endoscopic CO_2_ laser excision of the scar tissue by every laryngologist in our institution. The procedure was performed under general anesthesia with high-frequency positive pressure ventilation (HFPPV, Monsoon™ ventilation, Acutronic Medical Systems AG, Fabrik im Schiffli, CH-8816, Hirzel, Switzerland) through a steel, laser-resistant catheter. Stenosis was then either vaporized or divided with radial incisions through suspension microlaryngoscopy, depending on the nature of the cicatrix and its craniocaudal length.

During 2012, Superimposed High-Frequency Jet Ventilation (SHFJV^®^, Twinstream™, Mariannengasse 17, 1090 Wien, Austria) was introduced at our institution as a promising method for airway surgery. Concurrently, the absence of a ventilation catheter in the trachea favored the switch of our surgical approach from CO_2_ laser excisions to balloon dilatation of the stenotic part of the airway, which became the surgical method of choice by the end of that year and has exclusively been used since. Through suspension laryngoscopy under general anesthesia with SHJV^®^, a balloon catheter is advanced in the airway and gently dilates the stenotic part of the airway, following radial incisions with cold steel if appropriate. An INSPIRA AIR^®^ Balloon Dilatation System (Acclarent, Inc., 33 Technology Drive Irvine, CA 92618, USA) sized 14 mm at 10 atm pressure was used until 2017. It was then substituted by Continuous Radial Expansion™ balloons (Boston Scientific Corporation, 300 Boston Scientific Way, Marlborough, MA 01752, USA) for dilations of up to 15 mm at 8 atm pressure in females and 18 mm at 7 atm pressure in males. The pressure was applied during a short period of apnea aiming for a total of three-to-four dilatation attempts, with a duration between 1 and 2 min or until the patient started desaturating, and up to the maximum possible balloon expansion.

### Data collection

This sharp switch in the surgical approach of treating SGS in our department generated the two patient groups we utilized in this study: the cohort of patients treated with CO_2_ laser excisions (CLC) between 1 January 2006 and 31 December 2011, and the cohort of patients treated with balloon dilatation (BDC) between 1 January 2014 and 31 December 2019. The period from 1 January 2012 to 31 December 2013 was considered an adaptation period for both the surgeons and the anesthesiology staff to acquaint themselves with the novel techniques.

The follow-up time for both cohorts was set to 3 years postoperatively. The natural history of the disease after an endoscopic procedure is commonly implicated with a recurrence. In our study, this was defined as significant dyspnea requiring a new surgical treatment when assessed clinically with laryngotracheoscopy by an airway surgeon. Thus, the primary outcome of the study was determined as the time interval from the first surgery until the repeat surgery at recurrence (if it occurred), and the endpoints were a recurrence-free status at 3 years postoperatively or a surgical procedure for recurrence within the follow-up period. Demographic data extracted from the patients’ records included sex, age, time to SGS diagnosis, body mass index (BMI), SGS etiology, smoking history, the presence of diagnosed or self-reported GERD, and tracheal trauma from previous history of tracheostomy at any age or intubation within 2 years prior to the date of diagnosis. Other conditions registered from the patients’ records were diabetes, conditions of the lower airway or the lungs, and cardiovascular comorbidities, including ischemic heart disease, heart failure, arrhythmia, or cerebrovascular condition.

### Statistical analysis

A power calculation was made prior to performing the statistical analysis. A total of 72 patients were required to have an 80% chance of detecting a reduction in the recurrence rate from 80% in the CLC group to 50% in the BDC at 3 years postoperatively, which was significant at the 5% level [10, 11]. Continuous variables were analyzed by the Mann‒Whitney *U* test and are presented as medians and the 25th-to-75th percentiles, whereas categorical variables were analyzed by the Chi-square test or Fisher’s exact test when appropriate and are presented as numbers and percentages.

We visualized time to recurrence with the Kaplan‒Meier (KM) method and presented it as cumulative recurrence risk (1-KM). All patients were followed up after the initial operation to the first reoperation or censored at 3 years. Cox proportional hazard models were applied, estimating hazard ratios (HRs) with 95% confidence intervals (CIs) to compare disease recurrence for the two treatment groups. Models were both crude and adjusted for sex, age (categorized as 18–39, 40–49, 50–59, and ≥ 60 years), cause of SGS, smoking, positive intubation history within 2 years prior to the initial SGS diagnosis, BMI according to the World Health Organization (WHO) classification (< 25 kg/m^2^ defined as normal weight, 25–29.9 kg/m^2^ defined as overweight, and ≥ 30 kg/m^2^ defined as obese), presence of self-reported or diagnosed GERD, and diabetes. Confounders were chosen prior to data analysis and in accordance with the previous studies [5, 11, 12]. The proportional hazard assumption was tested by the phtest command in STATA. A *p* value less than 0.05 was considered statistically significant. IBM^®^ SPSS^®^ Statistics, version 27 (IBM Corp. Armonk, NY, USA) and STATA release 17 (StataCorp. 2021. College Station, TX: StataCorp LLC.) were used for the statistical analysis.

### Ethics

This human study was performed in accordance with the Declaration of Helsinki Guidelines and was approved by the Ethics Review Board in Uppsala (diary number 2016/193) and the Swedish Ethical Review Authority (diary numbers 2020-05509 and 2022-02708-02). All adult participants provided written informed consent to participate.

## Results

The study population consisted of 19 patients in the CLC and 31 patients in the BDC. We excluded 16 patients in total: Eight of them were previously treated for SGS outside our inclusion period, 3 subjects were found to have multilevel stenosis engaging other parts of the airway (2 with glottic, 1 with bronchial stenosis), 3 cases had a damaged cricotracheal cartilaginous framework and were not appropriate for endoscopic treatment, and in 2 cases treated with CO_2_ laser, we could not establish contact and receive an informed consent.

Both groups had a similar mean time to diagnosis, yet the mean age at diagnosis was significantly lower in the BLC. The most predominant SGS cause was the idiopathic type, followed by trauma, in both cohorts, and none of the patients were tracheostomized at any age. Table 1 lists the demographic data and comorbidities of the study population at baseline. Only one patient presented with ischemic heart disease. None of them were diagnosed with conditions of the lower airway or the lungs, yet 7 patients had been prescribed steroid inhalers by general practitioners suspecting asthma prior to the diagnosis of SGS. No readmissions or other complications were observed postoperatively for either surgical technique. Because of the relatively small sample size, the SGS cause variable was converted into a binary variable to consolidate the regression analysis.

**Table 1:** Demographic data of the study population

	CO_2_ laser	Balloon dilatation	*p* Value
*N*	19	31	
Age at diagnosis—median (25th–70th percentile)	58.0 (50.8–63.5)	47.0 (38.0–63.0)	**0.033**
18–30 years old—*n* (%)	0 (0)	9 (29)	**0.014**
40–49 years old—*n* (%)	2 (10.5)	7 (22.6)	
50–59 years old—*n* (%)	7 (36.8)	5 (16.1)	
≥ 60 years old—*n* (%)	10 (52.6)	10 (32.3)	
Years undiagnosed—median (25th–70th percentile)	1.8 (1.0–3.0)	2.0 (1.0–5.0)	0.59
Sex—*n* (%)			
Male	0	4 (12.9)	0.28
Female	19 (100)	27 (87.1)	
Cause—*n* (%)			
Idiopathic	12 (63.2)	25 (80.6)	0.49
Traumatic	3 (15.6)	3 (9.7)	
GPA	1 (5.3)	2 (6.5)	
IgG_4_-mediated disease	1 (5.3)	1 (3.2)	
Rheumatoid arthritis	1 (5.3)	0	
Other vasculitis	1 (5.3)	0	
BMI—*n* (%)			
Underweight/normal weight (< 25 kg/m^2^)	6 (31.6)	13 (41.9)	0.36
Overweight (25–29.9 kg/m^2^)	8 (42.1)	7 (22.6)	
Obese (≥ 30 kg/m^2^)	5 (26.3)	11 (25.5)	
Smoking history—*n* (%)			
Never smoker	16 (84.2)	27 (87.1)	0.99
Ever smoker	3 (12.9)	4 (12.9)	
Intubation history within 2 years prior to initial diagnosis—*n* (%)			
Positive	4 (21.1)	5 (16.1)	0.72
Negative	15 (78.9)	26 (83.9)	
GERD—*n* (%)			
Positive	6 (31.6)	7 (22.6)	0.52
Negative	13 (68.4)	24 (77.4)	
Diabetes—*n* (%)			
Positive	2 (10.5)	4 (12.9)	0.99
Negative	17 (89.5)	27 (81.1)	
Hypertension—*n* (%)			
Positive	6 (31.6)	5 (16.1)	0.29
Negative	13 (68.4)	26 (83.9)	

Bold values are statistically significant (*p* < 0.05)

*SD* standard deviation, *IQR* interquartile range, *BMI* body mass index, *GPA* granulomatosis with polyangiitis, *GERD* gastroesophageal reflux disease

**Fig. 1:**
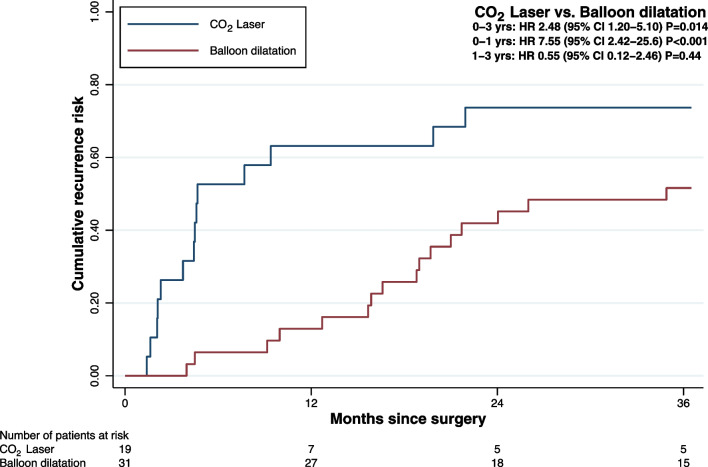
Kaplan‒Meier curves showing the cumulative risk for disease recurrence in patients undergoing CO_2_ laser (blue line) compared to balloon dilatation (red line) treatment for SGS

A total of 30 events were observed in the study population, 14 in the CLC and 16 in the BDC. The 3-year recurrence risk was 73.7% for the CLC (14 of 19 study subjects at risk) compared with 51.6% for the BDC (16 of 31 patients at risk, Fig. 1). As seen in the KM plot, the association between study groups was different during the 3-year follow-up, with a tendency for disease recurrence within the first year in the CLC and after the first year in the BDC. Since the proportional hazard assumption was violated, we modeled the group variable interact with the follow-up time (0–1 vs. 1–3 years) as an indicator variable to estimate time-dependent analysis [14, 15]. In the first year, the cumulative risk of recurrence was 63.2% for the CLC compared to 12.9% for the BDC, with a crude HR of 7.55 (95% CI 2.42–25.6, *p* < 0.001) and an adjusted HR of 33.0 (95% CI 6.57–166, *p* < 0.001). Among patients without a recurrence during the first year, the follow-up period from 1 to 3 years showed a crude HR of 0.55 (95% CI 0.12–2.46) and an adjusted HR of 1.85 (95% CI 0.32–10.8).

The adjusted model showed an elevated risk of recurrence in overweight (adjusted HR 3.44, 95% CI 1.16–10.2, *p* = 0.025) and obese patients (adjusted HR 7.11, 95% CI 2.19–23.0, *p* = 0.001) compared to normal-weight patients. The group of patients aged 40 years and below was also found to have a higher risk of recurrence (adjusted HR 8.18, 95% CI 1.43–46.8, *p* = 0.018) than the group of patients aged 50–59 years (Table 2).

**Table 2:** Time to recurrence evaluated with Cox regression to compare the CO2 laser treatment and balloon dilatation groups

	*N*	Outcomes	Crude (*n* = 50)		Adjusted (*n* = 50)	
		*n* (%)	HR (95% CI)	*p*	HR (95% CI)	*p*
Treatment^a^						
Balloon dilatation	31	16 (51.6)	Ref		Ref	
CO_2_ laser	19	14 (73.7)	2.48 (1.20–5.10)	**0.014**	8.02 (2.39–26.9)	**< 0.001**
0–1 Year follow-up^a^						
Balloon dilatation			Ref		Ref	
CO_2_ laser			7.55 (2.42–25.6)	**< 0.001**	33.0 (6.57–166)	**< 0.001**
1–3 year follow-up^a^						
Balloon dilatation			Ref		Ref	
CO_2_ laser			0.55 (0.12–2.46)	0.44	1.85 (0.32–10.8)	0.49
Gender						
Male	4	1 (25.0)	Ref		Ref	
Female	46	29 (63.0)	3.14 (0.43–23.1)	0.26	3.28 (0.36–29.7)	0.29
BMI						
Normal/underweight	19	7 (36.8)	Ref		Ref	
Overweight	15	11 (73.3)	2.73 (1.06–7.06)	**0.038**	3.44 (1.16–10.2)	**0.025**
Obese	16	12 (75.0)	3.52 (1.37–9.04)	**0.0089**	7.11 (2.19–23.0)	**0.0011**
Smoking history						
Never smoker	43	25 (58.1)	Ref		Ref	
Ever smoker	7	5 (71.4)	1.46 (0.56–3.82)	0.44	1.75 (0.44–6.94)	0.43
Intubation history						
Negative	41	24 (58.5)	Ref		Ref	
Positive	9	6 (66.7)	1.34 (0.55–3.29)	0.52	1.83 (0.44–7.60)	0.40
GERD						
Negative	37	21 (56.8)	Ref		Ref	
Positive	13	9 (69.2)	1.48 (0.67–3.23)	0.33	1.33 (0.33–5.36)	0.68
Cause						
Nonidiopathic	13	9 (69.2)	Ref		Ref	
Idiopathic	37	21 (56.8)	0.60 (0.27–1.32)	0.20	1.17 (0.22–6.27)	0.85
Age						
< 40	9	7 (77.8)	1.30 (0.45–3.72)	0.63	8.18 (1.43–46.8)	0.018
40–49	9	7 (77.8)	1.46 (0.51–4.20)	0.48	2.28 (0.54–9.65)	0.26
50–59	12	7 (58.3)	Ref		Ref	
≥ 60	20	9 (45.0)	0.71 (0.26–1.91)	0.50	0.73 (0.24–2.20)	0.58
Diabetes						
Negative	44	25 (56.8)	Ref		Ref	
Positive	6	5 (83.3)	2.09 (0.79–5.52)	0.14	2.26 (0.55–9.23)	0.26

Bold values are statistically significant (*p* < 0.05)

Patients are followed from the initial surgery up to 3 years

*HR* hazard ratio, *CI* confidence interval, *Ref* reference, *BMI* body mass index, *GERD* gastroesophageal reflux disease

^a^Treatment showed a nonproportional hazard and was evaluated as a time-dependent association by interaction with follow-up time (0–1 vs. 1–3 years

## Discussion

The primary findings of our study indicate a superiority of balloon dilatation compared to CO_2_ laser excisions in short-term disease recurrence, particularly within the first year postoperatively. Furthermore, patients who were overweight or obese or had a disease presentation at a younger age were independently found to have a statistically significant increased risk of SGS recurrence.

The diversity of surgical approaches in the endoscopic treatment of SGS, such as different dilation instruments (e.g., rigid endoscopes or inflatable balloons), scar excision instruments (e.g., cold steel or CO_2_ laser), and adjuvant therapies (e.g., mitomycin C or steroids), complicates the comparison of these procedures. The homogeneity of the two surgical techniques used in our study population facilitates, in essence, the comparison of the thermal effect of a CO_2_ laser excision with the cold tissue expansion of balloon dilatation, minimizing the confounding impact of different endoscopic treatments. This is reflected by the 51.6% risk of recurrence at 3 years for our BDC group, which is consistent with other studies investigating the outcomes of balloon dilatation without CO_2_ laser-assisted excisions [5, 6, 13]. There is indisputable evidence that open surgical techniques prevail regarding the durability of maintaining a patent airway without the need for tracheostomy or repeated surgery, eliminating dyspnea. However, they are associated with substantial perioperative risks (e.g., anastomotic complications or temporary tracheostomy). Postoperative morbidity, including poor voice outcomes or even an eventual delayed disease recurrence of up to 30% between 5 and 10 years postoperatively, cannot be overlooked [4, 6, 11, 16–18]. Thus, endoscopic treatment still has an important role in the treatment of SGS with its excellent convalescence and despite the higher recurrence rate when compared to open surgical procedures [5, 19].

Our results encourage the use of balloon dilatation instead of CO_2_ laser excisions considering the longer time to recurrence, since this is ultimately considered the natural course of the condition. We showed that there is a particular propensity for recurrence in the CLC during the first year postoperatively, whereas stenoses treated with balloon dilatation tend to recur during the second year of follow-up. Interestingly, there seems to be a trend of stability in the relapsing manner of the condition in both groups within the third year (73.7% for both the 2-year and 3-year recurrence risk for the CLC compared to 42.9% and 51.6%, respectively, for the BDC; Fig. 1). These findings could be considered in the context of preoperative patient counseling and the individual selection of an endoscopic treatment. The vigilant perspective of an exceptional increase in the incidence of laryngotracheal stenosis during the COVID-19 outbreak [20] led to a prioritized handling of patients with airway problems. Therefore, the treatment of patients with airway obstruction, in particular SGS recurrence, was never delayed.

Although stenoses related to iatrogenic trauma are regarded to be more prevalent [1, 21, 22], the profile of our study population matches the idiopathic type of the condition. Previously published studies have discussed potential environmental or hereditary factors related to the high prevalence of idiopathic SGS [12, 23–26]. However, this finding might also reflect the anticipative policy in our institution of striving for either tracheostomy in patients with expected prolonged intubation or prompt decannulation combined with noninvasive ventilation to minimize mucosal trauma and scarring predisposing for traumatic SGS. Furthermore, the idiopathic type consists predominantly of otherwise healthy, middle-aged, nonsmoking females experiencing symptoms of dyspnea for approximately 2 years before given the correct diagnosis of SGS [11, 13, 27]. An elevated BMI is also identified as a factor associated with disease recurrence [17, 28, 29]. This view is supported by our findings with a relatively low incidence of comorbidities, and HRs of 3.5 and 7.1 for overweight and obese patients, respectively, compared to normal or underweight patients. The large CIs observed apparently depend on our study’s small sample size.

The theory of a hormonal imbalance in perimenopausal females has been previously studied to explain the onset of idiopathic SGS in that age group. Estrogen receptors are thought to be expressed either unproportionally compared to progesterone receptors and more extensively in females with idiopathic SGS compared to patients with a nonidiopathic type of SGS [30, 31]. Moreover, there is evidence of an age-related elevation in peripheral estrogen formation occurring in adipose tissue [32]. Thus, being overweight or obese could potentially affect and complicate the hormonal equilibrium in premenopausal females contributing to the development of idiopathic SGS before menopause. Pregnancy-associated idiopathic SGS, although a rare entity, further supports the hypothesis of a hormonal origin or blossoming of symptoms in an established and occult stenosis due to the physiological vascular and respiratory changes of pregnancy [33]. These are concepts requiring further studies that could potentially explain the idiopathic prevalence in our cohort and the higher risk of recurrence in the fertile age group (18–39 years old) than in the peri- or postmenopausal age groups.

The major strength of our study is the segmentation of the inclusion period into nonoverlapping timeframes where the physicians in our department performed only one of two interventions, including a distinct learning period in between. In this manner, we sought to minimize performance bias, since nonrandom intervention assignment is a well-described disadvantage in all retrospective studies. Furthermore, only previously untreated patients with isolated stenosis of the subglottic region were included to eliminate the potential confounding effect of scar transformation by previous surgery and potential selection bias. Due to the relapsing nature of SGS and in conformity with results from previous reports [6, 11, 13], the follow-up time was set to 3 years for both cohorts, ensuring an equal and homogenous assessment of the survival analysis.

The absence of an objective and subjective severity grading of stenosis both before the initial intervention and at the clinical assessment upon recurrence is the main limitation of our study. An anatomical classification made by the surgeon was absent from the entire CLC, as neither the Cotton–Myer nor McCaffrey system had been used by the physicians in our department at that time. Although these scales have been widely proposed to assess SGS disease severity and prognosis, the former does not address the length and complexity of the lesion, and the latter does not justify the cross-sectional degree of stenosis [1]. Song et al. [34] showed the poor interrater reliability of a visual estimation in Cotton–Myer grading among physicians and further discussed the difficulty in identifying cricoid cartilage when assessing stenosis length endoscopically. Moreover, neither of the two systems correlates with functional airway assessment with spirometry, as shown by several studies [35–37]. Since there is evidence that several measurements of pulmonary function could be used in the diagnosis and postoperative monitoring of patients with SGS [34, 35], the lack of a preoperative functional evaluation with spirometry in our study population is considered another shortcoming of our study. Moreover, it would be interesting to quantify patient-experienced dyspnea using questionnaires specifically developed for upper airway obstruction [38, 39]. However, these data were missing from the entire CLC, since a routine assessment with spirometry and the validated Swedish version of the Dyspnea Index was not introduced as part of the preoperative workup in our department until 2016.

Our study indicates that balloon dilatation is superior to CO_2_ laser treatment in SGS patients, which is in conformity with several other retrospective studies [5, 6, 13]. Future prospective multicenter randomized control trials are recommended to achieve a sufficient sample size to further evaluate this evidence and examine the effect of adjuvant therapies and the associations of different patient-specific confounders predisposing patients to SGS recurrence.

## Conclusion

Endoscopic treatment for SGS with balloon dilatation is more favorable regarding short-term SGS recurrence compared to CO_2_ laser treatment, and patients with a younger age of SGS onset, overweight, or obesity showed a higher risk for SGS recurrence.

## Data Availability

The datasets generated during and/or analysed during the current study are available from the corresponding author on reasonable request.
